# Peptidyl arginine deiminase type IV (*PADI4*) haplotypes interact with shared epitope regardless of anti-cyclic citrullinated peptide antibody or erosive joint status in rheumatoid arthritis: a case control study

**DOI:** 10.1186/ar3051

**Published:** 2010-06-10

**Authors:** So-Young Bang, Tae-Un Han, Chan-Bum Choi, Yoon-Kyoung Sung, Sang-Cheol Bae, Changwon Kang

**Affiliations:** 1 Department of Rheumatology, Hanyang University Hospital for Rheumatic Diseases, 17 Hangdang-dong Seongdong-gu, Seoul 133-792, South Korea; 2 Department of Biological Sciences, Korea Advanced Institute of Science and Technology, 335 Gwahangno Yuseong-gu, Daejeon 305-701, South Korea

## Abstract

**Introduction:**

Anti-cyclic citrullinated peptide autoantibodies (anti-CCP) are the most specific serologic marker for rheumatoid arthritis (RA). Genetic polymorphisms in a citrullinating (or deiminating) enzyme, peptidyl arginine deiminase type IV (PADI4) have been reproducibly associated with RA susceptibility in several populations. We investigated whether *PADI4 *polymorphisms contribute to anti-CCP-negative as well as -positive RA, whether they influence disease severity (erosive joint status), and whether they interact with two major risk factors for RA, Human Leukocyte Antigen-DRB1 *(HLA-DRB1*) shared epitope (SE) alleles and smoking, depending on anti-CCP and erosive joint status.

**Methods:**

All 2,317 unrelated Korean subjects including 1,313 patients with RA and 1,004 unaffected controls were genotyped for three nonsynonymous (padi4_89, padi4_90, and padi4_92) and one synonymous (padi4_104) single-nucleotide polymorphisms (SNPs) in *PADI4 *and for *HLA-DRB1 *by direct DNA sequence analysis. Odds ratios (OR) were calculated by multivariate logistic regression. Interaction was evaluated by attributable proportions (AP), with 95% confidence intervals (CI).

**Results:**

A functional haplotype of the three fully correlated nonsynonymous SNPs in *PADI4 *was significantly associated with susceptibility to not only anti-CCP-positive (adjusted OR 1.73, 95% CI 1.34 to 2.23) but also -negative RA (adjusted OR 1.75, 95% CI 1.15 to 2.68). A strong association with both non-erosive (adjusted OR 1.62, 95% CI 1.29 to 2.05) and erosive RA (adjusted OR 1.62, 95% CI 1.14 to 2.31) was observed for *PADI4 *haplotype. Gene-gene interactions between the homozygous RA-risk *PADI4 *haplotype and SE alleles were significant in both anti-CCP-positive (AP 0.45, 95% CI 0.20 to 0.71) and -negative RA (AP 0.61, 95% CI 0.29 to 0.92). Theses interactions were also observed for both non-erosive (AP 0.48, 95% CI 0.25 to 0.72) and erosive RA (AP 0.46, 95% CI 0.14 to 0.78). In contrast, no interaction was observed between smoking and *PADI4 *polymorphisms.

**Conclusions:**

A haplotype of nonsynonymous SNPs in *PADI4 *contributes to development of RA regardless of anti-CCP or erosive joint status. The homozygous *PADI4 *haplotype contribution is affected by gene-gene interactions with *HLA-DRB1 *SE alleles.

## Introduction

Rheumatoid arthritis (RA) is a chronic inflammatory disease with a complex etiology that involves both genetic and environmental contributions; the pathogenesis of RA is still not fully understood. The genetic component of RA pathogenesis may account for up to 60%, and the Human Leukocyte Antigen (HLA) region in particular has shown the strongest genetic association with RA [1,2]. The *Human Leukocyte Antigen-DRB1 (HLA-DRB1) *shared epitope (SE) alleles are the most potent genetic risk factor for RA [2-5]. However, the effect of HLA polymorphisms accounts for only one-third of the overall genetic contribution observed. The peptidyl arginine deiminase type IV gene (*PADI4*) has been shown in several studies to be an additional RA susceptibility gene in Asians and in some Caucasian populations [6-11]. However, in several other Caucasian populations, no association has been found between *PADI4 *and RA [12-15].

Anti-cyclic citrullinated peptide autoantibodies (anti-CCP) are highly specific for RA [16-19], and the enzyme PADI4 deiminates certain arginine residues to citrullines in some proteins. The anti-CCP were detected more frequently in RA patients who were homozygous for an RA-susceptible haplotype of *PADI4*, and *PADI4 *messenger RNA (mRNA) of the susceptible haplotype was more stable than mRNA without it in a Japanese study [6]. We have previously demonstrated that increased serum levels of anti-CCP are associated with the RA-risk *PADI4 *haplotype in patients within 34 months of disease duration [20]. Accordingly, PADI4 may play a role in the citrullinating pathway of anti-CCP-positive RA pathogenesis. However, it has never been investigated whether the RA-risk haplotype of *PADI4 *contributes to the development of anti-CCP-negative RA as well.

Recently, it was reported that the association of *PADI4 *SNP with RA was restricted to patients with erosive disease (Steinbrocker score >II) in Caucasians [21]. However, their results were based on retrospective case-only analysis in a small sample size study.

Smoking is a major environmental risk factor for RA. It has been shown that smoking may trigger the RA immune reaction to citrullinated proteins and interact with SE alleles in development of RA [22-25]. Gene-environment interactions between SE alleles and smoking have been demonstrated in the development of anti-CCP-positive RA only [24,26-28]. However, we recently observed that SE alleles and smoking are associated with RA susceptibility in anti-CCP-positive as well as -negative RA [29]. A possible interaction between single SNP of *PADI4 *and smoking has been previously reported [30], but sample size examined was too small to fully clarify the gene-environment interactions. Therefore, this needs to be confirmed for other populations in large scale studies.

We studied a large case-control study to scrutinize the effects of *PADI4 *on joint destruction as an indicator of RA severity and synergic effects of *PADI4 *and major risk factors (SE alleles, smoking). First, we investigated whether *PADI4 *polymorphisms contribute differently to two subsets of RA categorized according to the presence and absence of anti-CCP or erosive joint state, respectively. Second, we assessed whether *PADI4 *polymorphisms interact with the *HLA-DRB1 *SE alleles in anti-CCP-positive/-negative RA as well as in non-erosive/erosive RA. Third, we investigated whether a gene-environment interaction occurs between *PADI4 *polymorphisms and smoking in a Korean population. Our findings provide insight into the pathogenic role of *PADI4 *in developing RA.

## Materials and methods

### Patients and controls

A total of 2,317 unrelated Korean subjects including 1,313 RA patients and 1,004 healthy controls, who were successfully genotyped for four exonic *PADI4 *SNPs and for *HLA-DRB1*, were included in this study among those recruited at Hanyang University Hospital for Rheumatic Diseases. All patients with RA met the American College of Rheumatology 1987 classification criteria [31]. Information about smoking status was obtained from 1,288 (98.1%) patients with RA and 991 (98.7%) controls in Korea. Information about direct smoking status was obtained using the same questionnaire given directly to the cases and controls by trained interviewers. Ever-smokers were defined as those individuals who had ever smoked cigarettes before the onset of RA. All patients with RA were classified into non-erosive (Steinbrocker stage I) and erosive (Steinbrocker stages II-IV) as a marker of RA severity at the time of enrollment [32]. Stage I RA was defined as the absence of destructive changes on radiographs, stage II RA as radiographic evidence of osteoporosis, with or without slight subchondral bone destruction or slight cartilage destruction, stage III RA as radiographic evidence of cartilage and bone destruction, subluxation, or ulnar deviation, and stage IV RA as fibrous or bony ankylosis.

The baseline characteristics of the RA patient and control subjects are shown in Table 1. The study was approved by the Institutional Review Board of Hanyang University Medical Center. Informed consent was obtained from all patients with RA and controls.

**Table 1:** Basic characteristics of patients with RA and control subjects*

	Controls (n = 1,004)	RA cases (n = 1,313)	anti-CCP-positive RA (n = 822)	anti-CCP-negative RA (n = 145)
Female, No. (%)	864 (86.1)	1,170 (89.1)	734 (89.3)	128 (88.3)
Age (mean ± SD years)	36.7 ± 12.5	51.8 ± 12.2	51.7 ± 11.8	50.5 ± 11.3
Onset age (mean ± SD years)	-	40.6 ± 12.4	40.5 ± 12.1	38.5 ± 11.9
Ever-smokers, No. (%)	134 (13.5)	197 (15.3)	119 (14.6)	26 (17.9)
Erosive disease (%)	-	1,071 (81.6)	682 (83.0)	113 (77.9)

* Except where indicated otherwise, values are the number (%). Among patients with rheumatoid arthritis (RA), 1,288 were evaluated for ever smokers, and 967 were evaluated for anti--cyclic citrullinated peptide antibodies (anti-CCP). Among control subjects, 991 were evaluated for ever smokers. Out of these subjects, 311 cases and 392 controls had been included in a previous study by Cha *et al. *[20].

### Genotyping of *PADI4* SNPs

Genomic DNA was extracted from peripheral blood mononuclear cells using the method of Miller *et al. *[33]. All RA patients and controls were genotyped for three nonsynonymous SNPs (padi4_89 (rs11203366), padi4_90 (rs11203367), and padi4_92 (rs874881)) and one synonymous SNP (padi4_104 (rs1748033)) in *PADI4*. Genotyping was performed using the MassARRAY system (Sequenom, San Diego, CA, USA) as described previously [8,20] with approval from the Institutional Review Board of Korea Advanced Institute of Science and Technology. The genotype distributions of cases and controls were found to be in Hardy-Weinberg equilibrium.

### Genotyping of *HLA-DRB1*

Allele-level genotypes of the *HLA-DRB1 *gene were obtained by conventional polymerase chain reaction sequence based typing method, as described previously [34]. Briefly, the polymorphic exon 2 of the *DRB1 *gene was amplified using group-specific primer sets, and was sequenced by automated cycle sequencing based on dye terminator chemistry using an ABI3100 Genetic Analyzer (Life Technologies, Carlsbad, CA, USA). The SE alleles were *0101, *0102, *0401, *0404, *0405, *0408, *0410, *1001, *1402, and *1406.

### Measurement of anti-CCP

The serum concentration of anti-CCP was measured for 967 RA patients (73.6% of the total 1,313 patients) using the ImmuLisa CCP ELISA test (IMMCO Diagnostics, Buffalo, NY, USA). Among them, 822 patients were positive (85.0%) with serum concentration levels of 25 units/ml or higher.

### Statistical analysis

The odds ratios (OR) and 95% confidence intervals (CI) of developing RA depending on anti-CCP or erosive joint status were calculated using multivariate logistic regression and adjusted for age and sex. The attributable proportions (AP) with 95% CI were also calculated to measure the gene-gene and gene-environment interactions according to anti-CCP and erosive joint status [28,35,36]. *P-*values less than 0.05 were considered significant. All statistical analyses were performed using SPSS software version 12.0 (SPSS Inc., Chicago, IL, USA). Inter-SNP linkage disequilibrium (LD) r^2 ^values among SNPs in *PADI4 *were calculated using the Haploview 4.0 program, and haplotypes were reconstructed using the Bayesian algorithm-based program Phase, version 2.1 [37]. Adjustment was also made for confounding factor by residential area. But, residential area had a negligible influence on our results and was not retained in final analyses.

## Results

### Confirmed association of *PADI4* with RA susceptibility

In this Korean population of 1,313 patients with RA and 1,004 healthy controls (Table 1), the minor alleles in four exonic SNPs of *PADI4 *were each shown to be associated with increased susceptibility to RA confirming previous association results obtained using a subset of this study population [8,20]. The three nonsynonymous SNPs (padi4_89, padi4_90 and padi4_92) in *PADI4 *were fully correlated (r^2 ^= 1.00) with each other in controls and RA patients, and constitute only two common haplotypes, ACC and GTG (with letters representing the nucleotides found at padi4_89, padi4_90, and padi4_92, respectively) in all subjects except only for three. Extremely rare haplotypes ACG (n = 4), and GCC (n = 1) were excluded from analysis. Carriage of padi4_89 (OR 1.41, 95% CI 1.26 to 1.59), padi4_90 (OR 1.41, 95% CI 1.26 to 1.59), and padi4_92 (OR 1.42, 95% CI 1.26 to 1.60) were associated with susceptibility to RA. The minor haplotype GTG carrying the minor RA-risk alleles had 1.42-fold increased odds of having RA than the major haplotype ACC carrying the major non-risk alleles, and GTG carriers had 1.64-fold increased odds versus the non-carriers having ACC/ACC. The fourth, synonymous SNP (padi4_104) in *PADI4 *was also associated with RA susceptibility (OR 1.33, 95% CI 1.18 to 1.50), but this allelic association was not statistically independent from the above haplotype association because this SNP was very highly correlated (r^2 ^= 0.78 approximately 0.79) with the nonsynonymous SNPs. In fact, the synonymous SNP association vanished (*P *= 0.31) when adjusted for the nonsynonymous SNPs. Therefore, the *PADI4 *association with RA was assessed only with the nonsynonymous-SNP haplotypes in the subsequent analyses.

Additionally, RA susceptibility associations of *HLA-DRB1 *SE alleles and smoking were confirmed in this population [29]. Although ORs for GTG haplotype and SE alleles were higher in this study with adjustment for smoking than in a previous study without such adjustment [8], the enrolled study population was suitable for analyzing the effects of RA-risk *PADI4 *haplotype, SE alleles, and smoking in stratification with anti-CCP positivity and for assessing their interactions.

### Association of *PADI4* with RA according to anti-CCP or erosive joint status

*PADI4 *haplotype GTG carriers had 1.73-fold and 1.75-fold increased odds of anti-CCP-positive and -negative RA, respectively, compared with the non-carriers having only ACC, after adjustment for age, sex, SE alleles, and smoking (Table 2). *HLA-DRB1 *SE carriers had 5.18-fold and 2.31-fold increased odds of anti-CCP-positive and -negative RA, respectively, versus the non-carriers. In addition, ever-smokers had 2.17-fold and 2.77-fold increased odds of anti-CCP-positive and -negative RA, respectively, versus the non-smokers. Accordingly, all three RA-risk factors, *PADI4 *GTG carriage, *HLA-DRB1 *SE alleles and smoking were each associated with susceptibility to not only anti-CCP-positive but also -negative RA.

**Table 2:** Association of *PADI4 *haplotypes, *HLA-DRB1 *SE alleles and smoking with susceptibility to anti-CCP-positive and -negative RA*

	Controls, No	All RA cases		anti-CCP-positive RA		anti-CCP-negative RA	
				
Subgroup		No.	OR (95% CI)	No.	OR (95% CI)	No.	OR (95% CI)
GTG-negative†	378	366	1	218	1	39	1
GTG-positive	625	945	1.64 (1.31 to 2.05)	602	1.73 (1.34 to 2.23)	106	1.75 (1.15 to 2.68)
SE-negative	644	429	1	226	1	71	1
SE-positive	359	882	4.31 (3.49 to 5.32)	594	5.18 (4.54 to 7.45)	74	2.31 (1.57 to 3.41)
Non-smoking	856	1,089	1	692	1	119	1
Smoking	134	197	2.28 (1.47 to 3.52)	119	2.17 (1.29 to 3.63)	26	2.77 (1.30 to 5.90)

* Values are the number of subjects. OR and 95% CI for GTG carriage versus non-carriage were adjusted for age, sex, SE alleles and smoking. OR and 95% CI for SE carriage versus non-carriage were adjusted for age, sex, GTG carriage and smoking. OR and 95% CI for smoking versus non-smoking were adjusted for age, sex, GTG carriage and SE alleles. RA, rheumatoid arthritis; anti-CCP, anti-cyclic citrullinated peptide autoantibody; OR, odds ratios; CI, confidence intervals.

† The letters in *PADI4 *haplotypes represent nucleotides in padi4_89, padi4_90, and padi4_92 SNPs, respectively. Extremely rare haplotypes ACG (n = 4), and GCC (n = 1) were excluded from analysis. Three subjects (two RA patients and one control) who carried ACC and a rare haplotype were excluded from the analysis and hence the GTG-negative subjects carried only ACC/ACC.

In 1,313 patients with RA, 81.6% (Steinbrocker stages II-IV) had erosive joint disease (stage I, 18.4%; stage II, 34.3%; stage III 31.3%; stage IV 15.9%). *PADI4 *haplotype GTG carriers had 1.62-fold and 1.62-fold increased odds of erosive and non-erosive RA, respectively (Table 3). *HLA-DRB1 *SE carriers also had 4.45-fold and 4.16-fold increased odds of erosive and non-erosive RA. In addition, ever-smokers had 2.01-fold and 3.83-fold increased odds of erosive and non-erosive RA. Accordingly, all three RA-risk factors, *PADI4 *GTG carriage, *HLA-DRB1 *SE alleles and smoking were each associated with susceptibility regardless of erosive joint status in multivariate analyses.

**Table 3:** Association of *PADI4 *haplotypes, *HLA-DRB1 *SE alleles and smoking with susceptibility to erosive and non-erosive RA*

	Controls, No	All RA cases		Erosive RA		Non-erosive RA	
				
Subgroup		No.	OR (95% CI)	No.	OR (95% CI)	No.	OR (95% CI)
GTG-negative†	378	366	1	299	1	67	1
GTG-positive	625	945	1.64 (1.31 to 2.05)	771	1.62 (1.29 to 2.05)	174	1.62 (1.14 to 2.31)
SE-negative	644	429	1	346	1	83	1
SE-positive	359	882	4.31 (3.49 to 5.32)	724	4.45 (3.55 to 5.57)	158	4.16 (2.97 to 5.83)
Non-smoking	856	1,089	1	904	1	185	1
Smoking	134	197	2.28 (1.47 to 3.52)	146	2.01 (1.25 to 3.25)	51	3.83 (2.02 to 7.27)

* Values are the number of subjects. OR and 95% CI for GTG carriage versus non-carriage were adjusted for age, sex, SE alleles and smoking. OR and 95% CI for SE carriage versus non-carriage were adjusted for age, sex, GTG carriage and smoking. OR and 95% CI for smoking versus non-smoking were adjusted for age, sex, GTG carriage and SE alleles. Erosive RA cases were classified as Steinbrocker scores II-IV. RA, rheumatoid arthritis; anti-CCP, anti-cyclic citrullinated peptide autoantibody; OR, odds ratios; CI, confidence intervals.

† Three subjects (two RA patients and one control) who carried ACC and a rare haplotype were excluded from the analysis and hence the GTG-negative subjects carried only ACC/ACC.

### Gene-gene interactions between *PADI4* haplotype and *HLA-DRB1* SE alleles

The strength of the interactions was measured by AP of the RA-developing risk (Table 4). In anti-CCP-positive/anti-CCP-negative RA, individuals carrying GTG and SE had a higher risk of developing RA than those carrying neither GTG nor SE. The risk of anti-CCP-positive RA (OR 11.63, 95% CI 7.73 to 17.51) associated with the presence of GTG and SE was much higher than that of anti-CCP-negative RA (OR 4.10, 95% CI 2.23 to 7.53). However, there were no statistically significant interactions between GTG carriage and SE carriage in anti-CCP-positive RA or anti-CCP-negative RA (Table 4).

**Table 4:** Interaction between *PADI4 *haplotypes and SE alleles in susceptibility to anti-CCP-positive and -negative RA*

	Controls, No	All RA cases		anti-CCP-positive RA		anti-CCP-negative RA	
				
Subgroup		No.	OR (95% CI)	No.	OR (95% CI)	No.	OR (95% CI)
GTG and SE carriage	1,003	1,311		820		145	
GTG-negative/SE-negative	245	109	1	50	1	18	1
GTG-negative/SE-positive	133	257	5.17 (3.57 to 7.48)	168	8.20 (5.23 to 12.85)	21	2.40 (1.18 to 4.89)
GTG-positive/SE-negative	399	320	1.89 (1.37 to 2.61)	176	2.32 (1.54 to 3.49)	53	1.80 (0.99 to 3.25)¶
GTG-positive/SE-positive†	226	625	7.46 (5.38 to 10.36)	426	11.63 (7.73 to 17.51)	53	4.10 (2.23 to 7.53)
Diplotype and SE carriage	1,003	1,311		820		145	
ACC/ACC/SE-negative	245	109	1	50	1	18	1
ACC/GTG/SE-negative	290	217	1.72 (1.22 to 2.42)	122	2.14 (1.39 to 3.30)	33	1.52 (0.81 to 2.88)¶
GTG/GTG/SE-negative	109	103	2.39 (1.56 to 3.66)	54	2.85 (1.68 to 4.85)	20	2.58 (1.24 to 5.37)
ACC/ACC/SE-positive	133	257	5.19 (3.58 to 7.51)	168	8.24 (5.25 to 12.92)	21	2.41 (1.18 to 4.92)
ACC/GTG/SE-positive	179	428	6.23 (4.42 to 8.77)	298	9.87 (6.47 to 15.06)	31	2.90 (1.50 to 5.60)
GTG/GTG/SE-positive‡	47	197	12.74 (8.03 to 20.23)	128	19.45 (11.32 to 33.42)	22	9.59 (4.39 to 20.98)

* OR and 95% CI were adjusted for age, sex, and smoking. RA, rheumatoid arthritis; anti-CCP, anti-cyclic citrullinated peptide autoantibody; OR, odds ratios; CI, confidence intervals.

† The attributable proportion (95% CI) due to interaction was 0.16 (-0.10 to 0.42) in anti-CCP-positive RA and 0.19 (-0.26 to 0.63) in anti-CCP-negative RA.

‡ The attributable proportion (95% CI) due to interaction was 0.45 (0.20 to 0.71) in anti-CCP-positive RA and 0.61 (0.29 to 0.92) in anti-CCP-negative RA.

¶Association was not significant (*P *= 0.05 and *P *= 0.20, respectively).

In addition, we analyzed the interaction between *PADI4 *diplotypes (rather than haplotype) and SE carriage. SE carriers homozygous for GTG haplotype were strongly associated with high risk of both anti-CCP-positive RA (OR 19.45, 95% CI 11.32 to 33.42) and anti-CCP-negative RA (OR 9.59, 95% CI 4.39 to 20.98) compared with SE non-carriers homozygous for the non-risk haplotype ACC. The GTG homozygote interacted with SE alleles to increase the risk of developing anti-CCP-positive RA (AP 0.45, 95% CI 0.20 to 0.71) as well as anti-CCP-negative RA (AP 0.61, 95% CI 0.29 to 0.92).

As shown in Table 5, the combination of homozygous *PADI4 *haplotype and HLA-DRB1 SE alleles significantly increased the risk in patients with RA (for non-erosive RA (OR 14.47, 95% CI 7.11 to 29.45); for erosive RA (OR 12.98, 95% CI 7.97 to 21.14)). The AP (95% CI) due to gene-gene interaction between homozygous *PADI4 *haplotype and SE alleles was 0.48 (0.25 to 0.72) in erosive disease and 0.46 (0.14 to 0.78) in non-erosive disease (Table 5).

**Table 5:** Interaction between *PADI4 *haplotypes and SE alleles in susceptibility to erosive and non-erosive RA *

	Controls, No	All RA cases		Erosive RA		Non-erosive RA	
				
Subgroup		No.	OR (95% CI)	No.	OR (95% CI)	No.	OR (95% CI)
GTG and SE carriage	1,003	1,311		1,070		241	
GTG-negative/SE-negative	245	109	1	89	1	20	1
GTG-negative/SE-positive	133	257	5.17 (3.57 to 7.48)	210	5.05 (3.41 to 7.47)	47	5.70 (3.04 to 10.67)
GTG-positive/SE-negative	399	320	1.89 (1.37 to 2.61)	257	1.80 (1.27 to 2.54)	63	2.11 (1.19 to 3.75)
GTG-positive/SE-positive†	226	625	7.46 (5.38 to 10.36)	514	7.52 (5.31 to 10.65)	111	7.73 (4.39 to 13.61)
Diplotype and SE carriage	1,003	1,311		1,070		241	
ACC/ACC/SE-negative	245	109	1	89	1	20	1
ACC/GTG/SE-negative	290	217	1.72 (1.22 to 2.42)	177	1.65 (1.14 to 2.38)	40	1.85 (1.01 to 3.41)
GTG/GTG/SE-negative	109	103	2.39 (1.56 to 3.66)	80	2.22 (1.41 to 3.50)	23	2.87 (1.40 to 5.86)
ACC/ACC/SE-positive	133	257	5.19 (3.58 to 7.51)	210	5.07 (3.42 to 7.51)	47	5.71 (3.05 to 10.70)
ACC/GTG/SE-positive	179	428	6.23 (4.42 to 8.77)	357	6.29 (4.37 to 9.03)	71	6.16 (3.41 to 11.11
GTG/GTG/SE-positive‡	47	197	12.74 (8.03 to 20.23)	157	12.98 (7.97 to 21.14)	40	14.47 (7.11 to 29.45)

* OR and 95% CI were adjusted for age, sex, and smoking. Erosive RA cases were classified as Steinbrocker scores II-IV. RA, rheumatoid arthritis; OR, odds ratios; CI, confidence intervals.

† The attributable proportion (95% CI) due to interaction was 0.19 (-0.06 to 0.43) in erosive RA and 0.11 (-0.27 to 0.48) in non-erosive RA.

‡ The attributable proportion (95% CI) due to interaction was 0.48 (0.25 to 0.72) in erosive RA and 0.46 (0.14 to 0.78) in non-erosive RA.

We also investigated interaction between homozygous *PADI4 *haplotype and SE alleles in non-erosive and erosive RA according to anti-CCP status. The AP was 0.47 (0.22 to 0.73) in erosive disease and 0.52 (0.16 to 0.87) in non-erosive disease among anti-CCP-positive RA. The attributable proportion was 0.53 (0.11 to 0.95) in erosive disease and 0.69 (0.32 to 1.06) in non-erosive disease among anti-CCP-negative RA, indicating that these interactions were statistically significant.

### No gene-environment interactions between *PADI4* and smoking

The combination of GTG carriage and smoking significantly increased the risk in patients with RA (for anti-CCP-positive (OR 3.61, 95% CI 1.98 to 6.57); for anti-CCP-negative RA (OR 4.59, 95% CI 1.91 to 11.04) (Supplementary table S1 in Additional file 1). The combination of the homozygous *PADI4 *haplotype and smoking significantly increased the risk in patients with RA (for anti-CCP-positive (OR 5.23, 95% CI 2.30 to 11.87); for anti-CCP-negative RA (OR 9.20, 95% CI 3.07 to 27.54).

However, no significant interactions were found between the GTG carriage and smoking for either anti-CCP-positive (AP 0.10, 95% CI -0.43 to 0.63) or anti-CCP-negative RA (AP -0.17, 95% CI -1.21 to 0.88) (Supplementary table S1 in Additional file 1). We also did not find any statistically significant interaction between the homozygous *PADI4 *haplotype and smoking in anti-CCP-positive RA (AP 0.23, 95% CI -0.37 to 0.83) and anti-CCP-negative RA (AP 0.18, 95% CI -0.72 to 1.08). The combination of the homozygous *PADI4 *haplotype and smoking increased the risk in patients with RA (for erosive RA (OR 4.22, 95% CI 1.95 to 9.17); for non-erosive RA (OR 8.59, 95% CI 3.27 to 22.56)) (Supplementary table S2 in Additional file 1). However, the gene-environment interaction between homozygous *PADI4 *haplotype and smoking was not observed in erosive RA (AP -0.16, 95% CI -1.04 to 0.72) and non-erosive RA (AP 0.27, 95% -0.43 to 0.97).

## Discussion

The most significant finding of this study is that *PADI4 *polymorphisms are associated with RA susceptibility, regardless of anti-CCP as well as erosive joint status. Moreover, significant gene-gene interactions between homozygous *PADI4 *GTG haplotype and *HLA-DRB1 *SE alleles were observed for developing anti-CCP-positive and -negative RA. Interestingly, we also observed gene-gene interactions in patients with non-erosive and erosive RA. An additional finding is the lack of gene-environment interaction between *PADI4 *polymorphisms and smoking. Our findings suggest that homozygous *PADI4 *GTG haplotype influences RA regardless of joint destruction, and exerts more significant effects on developing RA through interaction with SE alleles.

Several studies and meta-analyses have confirmed the strong association between *PADI4 *and RA in Asian populations [6-9, 38, 39]. In German and French populations, a weak association between *PADI4 *and RA was observed [10,11]. However, several studies using Caucasian populations have yielded conflicting findings [12-15] and it has not yet been demonstrated how the *PADI4 *polymorphisms influence RA susceptibility. Suzuki *et al. *[6] proposed that a susceptible *PADI4 *haplotype had significantly increased mRNA stability and half-life compared with a non-susceptibility reference haplotype, and they reported that RA-risk *PADI4 *haplotype homozygosity was associated with the presence of anti-CCP. Later, it was shown that anti-CCP levels were significantly higher in individuals homozygous for the *PADI4 *risk haplotype [6,40]. Several investigators have speculated that certain *PADI4 *polymorphisms would enhance citrullination and decrease tolerance for citrullinated proteins, which could lead to the production of anti-CCP and the development of RA [6,40]. However, the inconsistent associations between *PADI4 *polymorphisms and the presence or levels of anti-CCP [6,10,11,15,20] raised a question about this hypothesis. In this study, we demonstrated that *PADI4 *polymorphisms are significantly associated with anti-CCP-positive and -negative RA. Accordingly, the *PADI4 *gene is more likely to play an important role in another citrullination pathway than its role in anti-CCP formation.

In a recent study, B Hoppe *et al. *[21] performed PADI4 effects on erosive RA in investigation of 373 patients, with non-erosive patients as controls. Interestingly, they found the association of *PADI4 *SNP with RA was restricted to only patients with joint destruction. However, we also observed that the combination of *PADI4 *genes and SE alleles increased the risk of developing non-erosive RA as well, which is a result that has not been shown previously. Our results suggest that *PADI4 *gene is linked to the susceptibility of RA regardless of RA severity, such as erosive joint status. This discrepancy may be due to differences in sample size and the design of the study. Our findings are based on a relatively large size and case-control study, and we think that it might represent a better estimate of results from the risk factors.

Another mechanism proposed for RA association of *PADI4 *is that *PADI4 *polymorphisms may interact with an environmental factor, smoking, via citrullinated proteins, resulting in the development of RA. However, the interaction between smoking and *PADI4 *polymorphisms has not been confirmed, although a possible interaction between only single *PADI4 *SNP and smoking has been previously reported [30]. No significant interaction was observed between RA-risk *PADI4 *haplotype and smoking in this population of Koreans. The number of individuals in our study is fairly large, but the number of smokers with anti-CCP-negative RA is relatively small. This may make conclusions difficult, so additional larger-scale studies need to be performed.

We previously reported that *PADI4 *SNPs and *HLA-DRB1 *SE alleles had additive effects in terms of the risk of developing RA, although no significant gene-gene interaction was shown between *PADI4 *SNPs and SE alleles because of the small sample size [8]. In this large population, significant interaction was detected between *PADI4 *risk haplotype homozygotes and SE alleles in both anti-CCP-positive and -negative RA. These results suggest that the homozygous *PADI4 *risk haplotype contribution to RA pathogenesis may be influenced by *HLA-DRB1 *SE alleles. These results conflict with a recent finding of no interaction between one *PADI4 *SNP and SE alleles in a large UK Caucasian population [15]. The *PADI4 *polymorphism and SE alleles appear to vary according to ethnicity. This discrepancy between Koreans and Caucasians could be attributed to genetic heterogeneity of RA from ethnic differences. Accordingly, these conflicting results of interaction may be explained by differences in target *PADI4 *SNP (padi4_89, padi4_90, padi4_92 vs padi4_94) or by differences in the major RA-susceptible SE alleles (for example, *0405 vs *0401) between Korean and Caucasian populations [41,42].

## Conclusions

The *PADI4 *gene contributed significantly to the development of RA, regardless of anti-CCP or erosive joint status. Strong gene-gene interactions between homozygous *PADI4 *haplotype and SE alleles occur in anti-CCP-positive/negative as well as erosive/non-erosive RA. Therefore, the *PADI4 *gene appears to play an important pathogenic role in all subsets of RA.

## Abbreviations

anti-CCP: anti-cyclic citrullinated peptide antibodies; AP: attributable proportions; CI: confidence intervals; HLA: Human Leukocyte Antigen; *HLA-DRB1: Human Leukocyte Antigen-DRB1*; LD: linkage disequilibrium; OR: odds ratios; *PADI4*: peptidyl arginine deiminase type IV gene; RA: rheumatoid arthritis; SE: shared epitope; SNP: single nucleotide polymorphism.

## Competing interests

The authors declare that they have no competing interests.

## Authors' contributions

Drs. Bang and Han contributed equally to this work. Drs. Bae and Kang had full access to all of the data in this study and take responsibility for the integrity of the data and the accuracy of the data analysis. Bang and Bae participated in the study design, acquisition of data, analysis and interpretation of data, statistical aspects, and drafting of the manuscript. Han and Kang contributed to data analysis and the drafting of the manuscript. Choi and Sung contributed through the assessment of clinical aspects. All authors read and approved the final manuscript.

## Supplementary Material

Additional file 1 **Supplementary tables S1-S2**. Supplementary table S1: Interaction between *PADI4 *haplotypes and smoking in susceptibility to anti-CCP-positive and -negative RA. Supplementary table S2: Interaction between *PADI4 *haplotypes and smoking in susceptibility to erosive and non-erosive RA. Click here for file
