# Supplementary material for: Peptidyl arginine deiminase type IV (PADI4) haplotypes interact with shared epitope regardless of anti-cyclic citrullinated peptide antibody or erosive joint status in rheumatoid arthritis: a case control study
Source: Arthritis Res Ther. 2010 Jun 10;12(3):R115. doi: 10.1186/ar3051 (PMC2911908; doi:10.1186/ar3051)
Supplement: Additional file 1 — Supplementary tables S1-S2. Supplementary table S1: Interaction between PADI4 haplotypes and smoking in susceptibility to anti-CCP-positive and -negative RA. Supplementary table S2: Interaction between PADI4 haplotypes and smoking in susceptibility to erosive and non-erosive RA. [file ar3051-S1.DOC]

Supplementary Table 1. Interaction between *PADI4* haplotypes and smoking in susceptibility to anti-CCP-positive and -negative RA*

|  | Controls, | All RA cases | |  | anti-CCP-positive RA cases | |  | anti-CCP-negative RA cases | |
| --- | --- | --- | --- | --- | --- | --- | --- | --- | --- |
| Subgroup | No. | No. | OR (95% CI) |  | No. | OR (95% CI) |  | No. | OR (95% CI) |
| GTG carriage and smoking | *n* = 990 | *n* = 1,286 |  |  | *n* = 811 |  |  | *n* = 145 |  |
| GTG-negative/non-smoking | 332 | 301 | 1 |  | 185 | 1 |  | 31 | 1 |
| GTG-negative/smoking | 43 | 57 | 3.02 (1.62−5.63) |  | 30 | 2.45 (1.17−5.12) |  | 8 | 4.01 (1.33−12.10) |
| GTG-positive/non-smoking | 524 | 788 | 1.73 (1.37−2.19) |  | 507 | 1.77 (1.35−2.32) |  | 88 | 1.90 (1.20−3.03) |
| GTG-positive/smoking† | 91 | 140 | 3.40 (2.05−5.66) |  | 89 | 3.61 (1.98−6.57) |  | 18 | 4.59 (1.91−11.04) |
| Diplotype and smoking | *n* = 990 | *n* = 1,286 |  |  | *n* = 811 |  |  | *n* = 145 |  |
| ACC/ACC/non-smoking | 332 | 301 | 1 |  | 185 | 1 |  | 31 | 1 |
| ACC/GTG/non-smoking | 393 | 546 | 1.50 (1.17−1.93) |  | 358 | 1.56 (1.17−2.08) |  | 56 | 1.52 (0.93−2.50) ¶ |
| GTG/GTG/non-smoking | 131 | 242 | 2.54 (1.83−3.53) |  | 149 | 2.55 (1.74−3.72) |  | 32 | 3.28 (1.82−5.92) |
| ACC/ACC/smoking | 43 | 57 | 3.04 (1.63−5.67) |  | 30 | 2.44 (1.17−5.12) |  | 8 | 4.04 (1.33−12.30) |
| ACC/GTG/smoking | 69 | 89 | 2.92 (1.66−5.15) |  | 58 | 3.03 (1.57−5.88) |  | 8 | 2.86 (1.01−8.14) |
| GTG/GTG/smoking‡ | 22 | 51 | 4.75 (2.33−9.69) |  | 31 | 5.23 (2.30−11.87) |  | 10 | 9.20 (3.07−27.54) |

* OR and 95% CI were adjusted for age, sex, and SE alleles. RA = rheumatoid arthritis; anti-CCP = anti-cyclic citrullinated peptide autoantibody; OR = odds ratios; CI = confidence intervals.

† The attributable proportion (95% CI) due to interaction was 0.10 (−0.43 to 0.63) in anti-CCP-positive RA and −0.17 (−1.21 to 0.88) in anti-CCP-negative RA.

‡ The attributable proportion (95% CI) due to interaction was 0.23 (−0.37 to 0.83) in anti-CCP-positive RA and 0.18 (−0.72 to 1.08) in anti-CCP-negative RA.

¶ Association was not significant (*P* = 0.10).

Supplementary Table 2. Interaction between *PADI4* haplotypes and smoking in susceptibility to erosive and non-erosive RA *

|  | Controls, | All RA cases | |  | Erosive RA cases | |  | Non-erosive RA cases | |
| --- | --- | --- | --- | --- | --- | --- | --- | --- | --- |
| Subgroup | No. | No. | OR (95% CI) |  | No. | OR (95% CI) |  | No. | OR (95% CI) |
| GTG carriage and smoking | *n* = 990 | *n* = 1,286 |  |  | *n* = 1050 |  |  | *n* = 236 |  |
| GTG-negative/non-smoking | 332 | 301 | 1 |  | 249 | 1 |  | 52 | 1 |
| GTG-negative/smoking | 43 | 57 | 3.02 (1.62−5.63) |  | 44 | 2.89 (1.47−5.68) |  | 13 | 4.00 (1.60−10.03) |
| GTG-positive/non-smoking | 524 | 788 | 1.73 (1.37−2.19) |  | 655 | 1.74 (1.35−2.23) |  | 133 | 1.64 (1.11−2.42) |
| GTG-positive/smoking† | 91 | 140 | 3.40 (2.05−5.66) |  | 102 | 2.90 (1.66−5.05) |  | 38 | 6.14 (2.91−12.97) |
| Diplotype and smoking | *n* = 990 | *n* = 1,286 |  |  | *n* = 1050 |  |  | *n* = 236 |  |
| ACC/ACC/non-smoking | 332 | 301 | 1 |  | 249 | 1 |  | 52 | 1 |
| ACC/GTG/non-smoking | 393 | 546 | 1.50 (1.17−1.93) |  | 459 | 1.52 (1.17−1.98) |  | 87 | 1.34 (0.88−2.04) ¶ |
| GTG/GTG/non-smoking | 131 | 242 | 2.54 (1.83−3.53) |  | 196 | 2.52 (1.78−3.56) |  | 46 | 2.74 (1.64−4.57) |
| ACC/ACC/smoking | 43 | 57 | 3.04 (1.63−5.67) |  | 44 | 2.91 (1.48−5.73) |  | 13 | 3.97 (1.58−9.97) |
| ACC/GTG/smoking | 69 | 89 | 2.92 (1.66−5.15) |  | 65 | 2.44 (1.31−4.53) |  | 24 | 5.16 (2.25−11.83) |
| GTG/GTG/smoking‡ | 22 | 51 | 4.75 (2.33−9.69) |  | 37 | 4.22 (1.95−9.17) |  | 14 | 8.59 (3.27−22.56) |

* OR and 95% CI were adjusted for age, sex, and SE alleles. RA = rheumatoid arthritis; OR = odds ratios; CI = confidence intervals.

† The attributable proportion (95% CI) due to interaction was −0.30 (−1.03 to 0.42) in erosive RA and 0.20 (−0.40 to 0.80) in non-erosive RA.

‡ The attributable proportion (95% CI) due to interaction was −0.16 (−1.04 to 0.72) in erosive RA and 0.27 (−0.43 to 0.97) in non-erosive RA.

¶ Association was not significant (*P* = 0.17).
